# High-efficiency and broadband asymmetric spin–orbit interaction based on high-order composite phase modulation

**DOI:** 10.1515/nanoph-2024-0344

**Published:** 2024-09-06

**Authors:** Yuzhong Ou, Yan Chen, Fei Zhang, Mingbo Pu, Mengna Jiang, Mingfeng Xu, Yinghui Guo, Chaolong Feng, Ping Gao, Xiangang Luo

**Affiliations:** National Key Laboratory of Optical Field Manipulation Science and Technology, Chinese Academy of Sciences, State Key Laboratory of Optical Technologies on Nano-Fabrication and Micro-Engineering, Institute of Optics and Electronics, Chengdu 610209, China; College of Materials Sciences and Opto-Electronic Technology, University of Chinese Academy of Sciences, Beijing 100049, China; State Key Laboratory of Optical Technologies on Nano-Fabrication and Micro-Engineering, Chinese Academy of Sciences, Institute of Optics and Electronics, Chengdu 610209, China

**Keywords:** asymmetric spin-orbit interaction, generalized geometric phase, all-metallic structure, metasurface

## Abstract

Asymmetric spin–orbit interaction (ASOI) breaks the limitations in conjugate symmetry of traditional geometric phase metasurfaces, bringing new opportunities for various applications such as spin-decoupled holography, imaging, and complex light field manipulation. Since anisotropy is a requirement for spin–orbit interactions, existing ASOI mainly relies on meta-atom with C1 and C2 symmetries, which usually suffer from an efficiency decrease caused by the propagation phase control through the structural size. Here, we demonstrate for the first time that ASOI can be realized in meta-atoms with rotational symmetry ≥3 by combining the generalized geometric phase with the propagation phase. Utilizing an all-metallic configuration, the average diffraction efficiency of the spin-decoupled beam deflector based on C3 meta-atoms reaches ∼84 % in the wavelength range of 9.3–10.6 μm, which is much higher than that of the commonly used C2 meta-atoms with the same period and height. This is because the anisotropy of the C3 metasurface originates from the lattice coupling effect, which is relatively insensitive to the propagation phase control through the meta-atom size. A spin-decoupled beam deflector and hologram meta-device were experimentally demonstrated and performed well over a broadband wavelength range. This work opens a new route for ASOI, which is significant for realizing high-efficiency and broadband spin-decoupled meta-devices.

## 1 Introduction

In optics and electromagnetics, circularly polarized light undergoes spin–orbit interaction (SOI) in anisotropic structures, and the cross-polarized light acquire a Pancharatnam–Berry (PB) geometric phase *ϕ* = 2*σθ* related to the rotation angle of the structure [1], [2], [3], [4], where *θ* is the orientation angle of the anisotropic structure, and *σ* = ±1 indicates left- and right-handed circular polarization (LCP and RCP) [5], [6], [7], [8]. PB phase metasurfaces have attracted great interest due to their simple and robust phase control scheme [9], [10]. In the past decades, geometric phase metasurfaces have been widely applied to fields such as focusing [11], holography [12], orbital angular momentum manipulation [13], etc. [14], [15]. However, pure geometric phase metasurfaces only support symmetric SOI, and there is a fundamental challenge of conjugate symmetry between the emergent light fields corresponding to LCP and RCP. This leads to limited manipulation dimensions and degrees of freedom [13], [16], [17], [18]. Inspired by the extraordinary Young’s interference in double slits with different widths [4], [19] and catenary optics [13], [20], we found that by merging geometric and propagation phase, the symmetry of photonic SOI of the composite phase metasurface can be broken, and asymmetric photonic spin–orbit interactions can be realized [21], [22], [23]. Asymmetric spin–orbit interaction (ASOI) opens up new possibilities for multifunctional meta-devices, complex vector optical field manipulation, and other applications [24]–[28].

Since anisotropy is a requirement for SOI in linear optics, existing researches on ASOI are largely limited to C1 and C2 rotationally symmetric meta-atoms. In recent years, meta-atoms with rotational symmetry ≥3 in symmetry-incompatible lattices have been found to generate generalized PB phase through SOI [29], [30], [31], [32], which exceed twice the rotation angle of the elements and provide new physics for disruptive applications. This indicates that the lattice coupling effect can induce anisotropy in high-fold rotationally symmetric elements. These evanescent coupling can be described by catenary optical fields [19]. However, only symmetric SOI has been realized in these structures. Another problem for ASOI is that the control of the propagation phase through the structural size will reduce the conversion efficiency, owing to the anisotropy of the meta-atom is usually sensitive to the structural size [33], [34], [35].

In this paper, we demonstrate ASOI in C3 rotationally symmetric meta-atoms by merging the generalized geometric phase and propagation phase. By designing a reflective all-metallic structure, the simulated conversion efficiencies for the C3 composite phase metasurface exceed 90 % at the central wavelength of 10 μm. The efficiency changes caused by the structural parameter changes for propagation phase modulation of C3 meta-atoms are much lower than those of C2 meta-atoms. As a proof of concept, a spin-decoupled beam deflector and hologram meta-device based on C3 meta-atoms were experimentally demonstrated, which agree well with theoretical expectations. This work provides a new approach for the realization of ASOI, which is of certain significance for realizing high-performance and easy-to-fabricate spin-decoupled meta-devices.

## 2 Results and discussion

### 2.1 Principles of ASOI based on high-order composite phase manipulation

Traditional composite phase metasurface for achieving ASOI is generally realized by combining the PB geometric phase with the propagation phase. This paper proposes a high-order composite phase metasurface by combining the generalized PB phase with the propagation phase. The generalized PB phase originates from the lattice coupling effect, which is caused by high-fold rotationally symmetric meta-atoms in a symmetrically incompatible lattice. The generalized PB phase is several times the traditional PB phase. For the *m*-fold (*m* ≥ 3) rotationally symmetric meta-atoms in a square lattice, the generalized geometric phase can be expressed as *ϕ* = ±*σmθ* (*m* is even) or *ϕ* = ±*σ*2*mθ* (*m* is odd), where the sign ± is related to the rotation direction of the principal axis [30].

**Figure 1:**
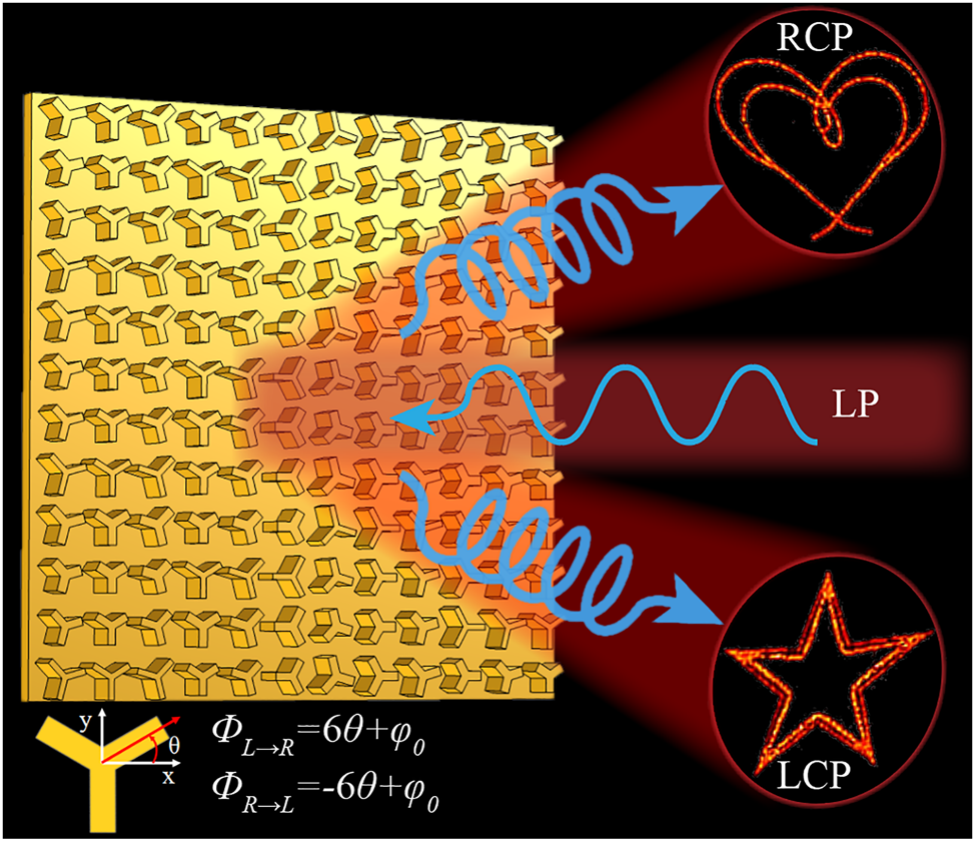
Schematic diagram of ASOI based on high-order composite phase manipulation.

Taking C3 meta-atom as an example, the generalized geometric phase is 6*σθ*. Combined with the propagation phase *ϕ*_0_ controlled by geometric size of the meta-atom, the high-order composite phase for LCP and RCP incidences can be written as:

(1) $$\phi_{L\to R}=6\theta +\phi_{0}$$

(2) $$\phi_{R\to L}=-6\theta +\phi_{0}$$

where *ϕ*_*L*→*R*_ and *ϕ*_*R*→*L*_ represent the phase shifts of the reflected RCP and LCP lights, respectively. Spin-decoupled manipulation can be achieved when the propagation phase *ϕ*_0_ covers the full 360° range. As illustrated in Figure 1, different holographic images can be generated for LCP and RCP output under linearly polarized (LP) incidence. For the given phase profiles *ϕ*_*L*→*R*_ and *ϕ*_*R*→*L*_, the required rotation angle *θ* and propagation phase *ϕ*_0_ can be calculated by:

(3) $$\theta =\frac{1}{12}\left(\phi_{L\to R}-\phi_{R\to L}\right)$$

(4) $$\theta =\frac{1}{2}\left(\phi_{L\to R}+\phi_{R\to L}\right)$$

### 2.2 Design of C3 composite phase metasurface and the comparison with commonly used C2 metasurface

**Figure 2:**
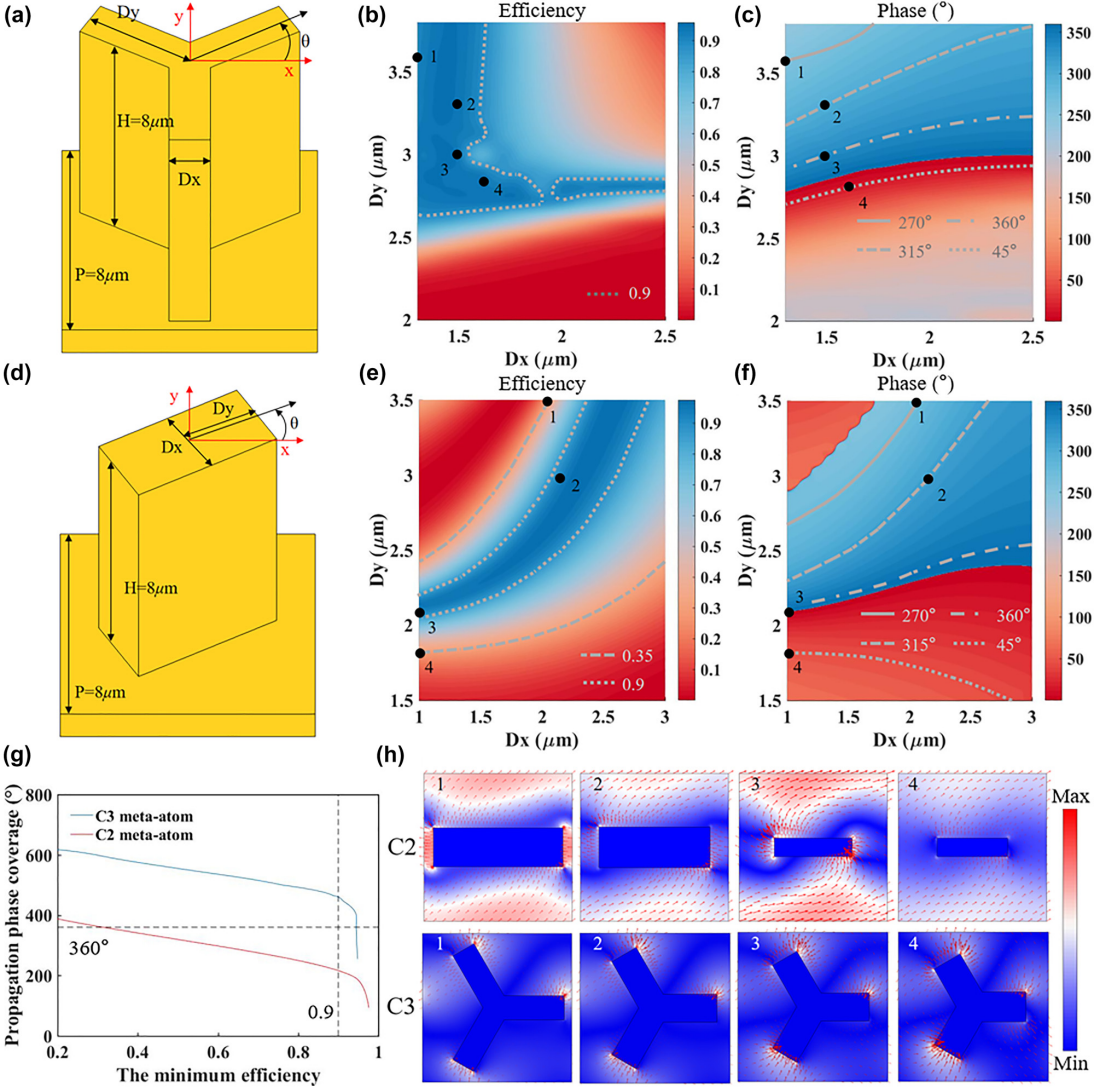
Design of C3 meta-atoms and comparison with C2 meta-atoms. (a) C3 structure model. Simulated (b) conversion efficiency and (c) propagation phase of C3 meta-atoms as a function of structural parameters. (d) C2 structure model. Simulated (e) conversion efficiency and (f) propagation phase of C2 meta-atoms as a function of structural parameters. (g) The propagation phase coverage of C3 and C2 structure as a function of the minimum conversion efficiency. (h) Simulated electric fields distribution and polarization vectors (red arrows) of C3 and C2 meta-atoms for RCP incidence.

Since all-metallic structure has higher performance in achieving high-efficiency and broadband generalized PB phase modulation ability than the metal-insulator-metal and all-dielectric schemes [29], this paper chooses a Y-shaped gold (Au) meta-atom with 3-fold rotational symmetry as the metasurface building block, as shown in Figure 2(a). The generalized geometric phase can be controlled by changing its in-plane rotation angle *θ*, and the propagation phase can be controlled by changing the arm length *Dy* or arm width *Dx* of the Y-shaped structure. Parameter scanning of the C3 structure was performed at the wavelength of 10 μm, with the simulated efficiency and phase shown in Figure 2(b) and (c). This paper selects 4 basic structures (black dots 1–4) and rotates each structure by 90° to form 4 new structures, to provide 8 discrete phase levels covering the full 360° range. The conversion efficiencies of 4 basic structures are 0.91, 0.94, 0.93, and 0.94, respectively. The absorption loss from the C3 meta-atom is about 0.05 at the wavelength of 10.2 μm (see Section S5 of Supplementary Material for details).

To demonstrate the advantages of the C3 structure, this paper also performs a parameter scanning of the C2 structure at the wavelength of 10 μm, as shown in Figure 2(d)–(f). For comparison, the C2 meta-atom has the same period, height, and linewidth range as the C3 structure mentioned above. To achieve full 360° propagation phase coverage for the 8 structures, 4 basic structures (black dots 1–4) covering approximately 180° phase range are selected, with the conversion efficiencies of 0.35, 0.95, 0.94 and 0.35, respectively. It can be seen that the efficiency changes with the structural variation for propagation phase modulation of C3 meta-atoms are much lower than those of C2 meta-atoms. Therefore, for all-metallic configurations, C3 meta-atom is a better choice to achieve high-efficiency and easy-to-fabricate ASOI meta-devices than C2 meta-atom.

To make a clearer comparison between C3 and C2 meta-atoms, we calculated their propagation phase coverage as a function of the minimum conversion efficiency in a selected set of structures, as shown in Figure 2(g). It can be seen that when the propagation phase coverage reaches 360°, the minimum efficiency among the selected C3 meta-atoms is about three times that of the selected C2 meta-atoms. The propagation phase coverage of the C3 meta-atom meets the 360° requirement when the minimum efficiency of the selected meta-atoms reaches 90 %, while the propagation phase coverage range of the C2 meta-atom is about half of that of the C3 meta-atom and does not meet the requirement.

Figure 2(h) shows the electric fields distribution of the selected C3 and C2 meta-atoms for RCP incidence. It can be seen that the electric field intensity and shape distributions of the C3 meta-atoms only show minimal changes when altering the structural parameters, while those of the C2 meta-atoms exhibit significant variations. This is because the anisotropy of the C3 meta-atoms originates from the lattice coupling effect, which makes it less sensitive to the changes of the meta-atom shapes compared to the C2 meta-atoms. Especially, when the length and width of the C2 meta-atom are equal, the C2 rotational symmetry will transform into the C4 rotational symmetry, causing the polarization conversion to disappear. This paper also conducts a comparative analysis between the C3 meta-atom and the C5 meta-atom. The C3 meta-atom emerges as a superior option for the development of high-efficiency and easy-to-fabricate ASOI meta-devices when compared to the C5 meta-atom. This advantage is attributed to the relatively weak anisotropy of the C5 meta-atoms in a square lattice configuration, which consequently leads to suboptimal conversion efficiency (detailed discussion shown in Section S6, Supplementary Material).

**Figure 3:**
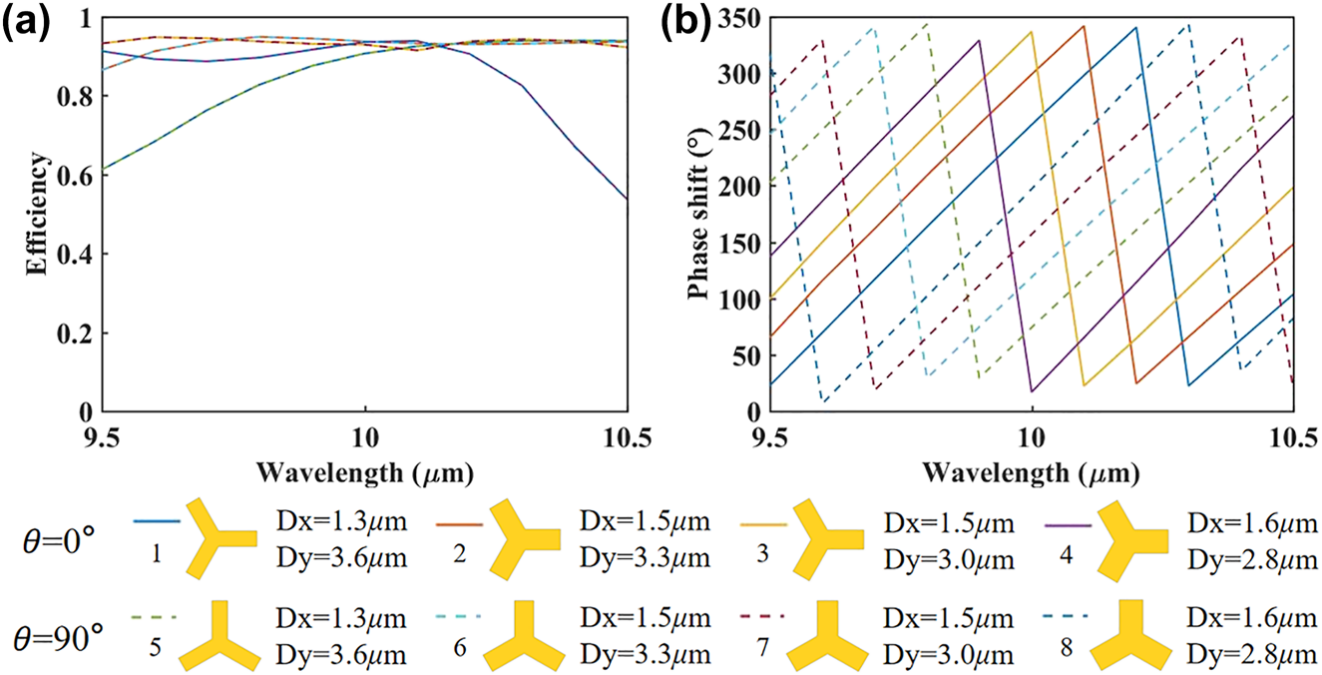
Simulation results of the unit structures for C3 composite phase metasurface. Simulated (a) conversion efficiency and (b) phase shift as a function of wavelength.

To verify the response bandwidth of the C3 meta-atoms, Figure 3(a) and (b) demonstrate the conversion efficiency and phase shift as a function of wavelength in the broadband range of 9.5–10.5 μm. It can be seen that the average efficiency of the selected structures exceeds ∼85 % in the wavelength range of 9.5–10.5 μm, and reaches a maximum of ∼94 % at 10 μm. The propagation phase of the 8 selected elements can cover 360° in a broad range of 9.5–10.5 μm, this enables them for broadband high-order composite phase modulations. Figure 3 also shows the detailed parameters for 8 selected structures of the C3 meta-atoms.

## 3 Experiments and characterization

### 3.1 Demonstration of the spin-decoupled beam deflector

**Figure 4:**
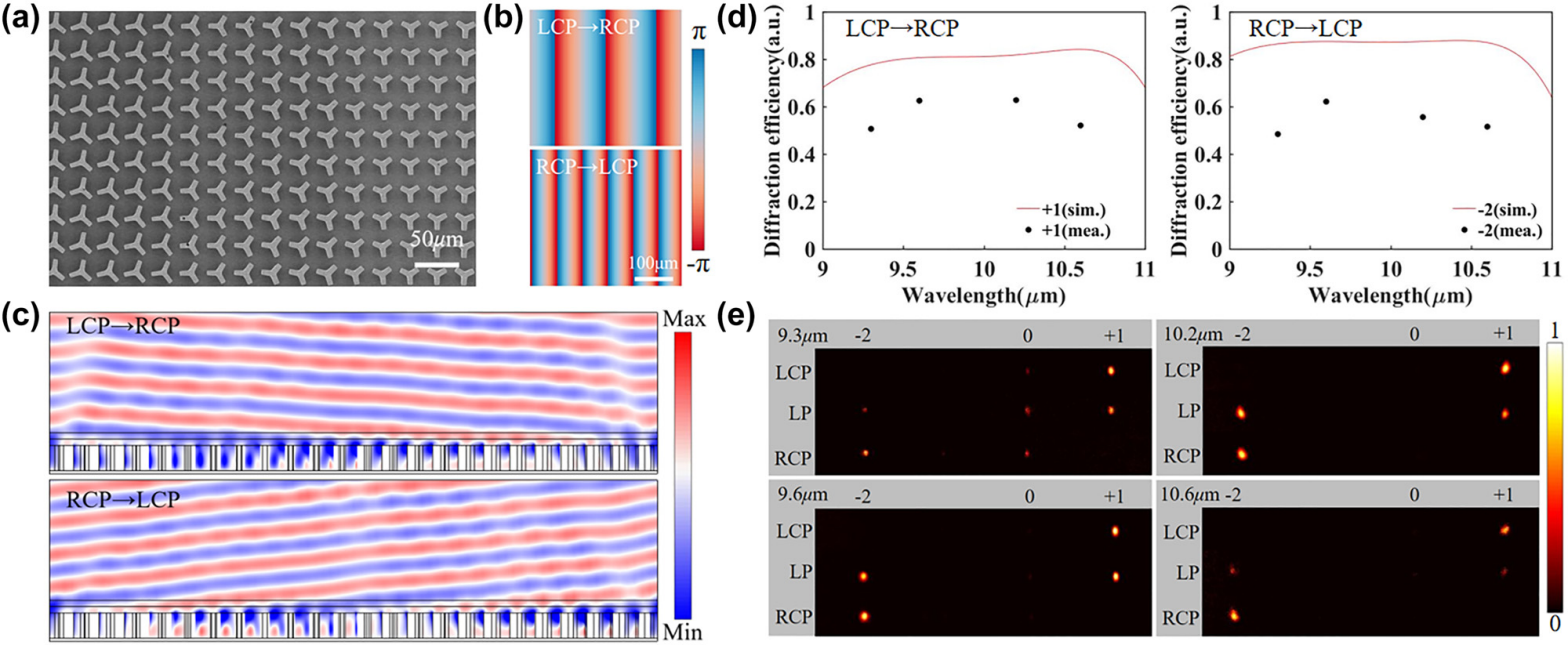
Spin-decoupled meta-deflector with C3 meta-atoms. (a) SEM image of the fabricated meta-deflector device. (b) The phase maps for the meta-deflector for LCP and RCP incidences. (c) Simulated *E*_cross_ field distributions of the metasurface at 10 μm for LCP and RCP incidences. (d) Simulated (sim.) and measured (mea.) diffraction efficiency of the C3 deflector under LCP and RCP illuminations. (e) Measured diffraction patterns of the deflector at different wavelengths under LCP, LP, and RCP illuminations.

A spin-decoupled beam deflector with C3 meta-atoms was designed and fabricated to demonstrate the high-efficiency and broadband spin-decoupled phase control ability. The SEM image of the fabricated deflector is shown in Figure 4(a) (detailed SEM images of the sample are shown in Supplementary Figure S1). The deflector consists of periodically arranged supercells, which contain 24 meta-atoms with different sizes and rotation angles. The phase gradients between adjacent meta-atoms are designed as 15° and 30° for LCP and RCP incidences, and the corresponding deflection angles are 3° and 6°, respectively. The phase maps are shown in Figure 4(b). Figure 4(c) shows the simulated cross-polarized electric fields *E*_cross_ under LCP and RCP incidence at 10 μm. The far-field intensity distribution at the central wavelength 10 μm is shown in Supplementary Figure S2. It can be seen that the cross-polarized RCP or LCP lights are deflected to the opposite directions, and the deflection angle for LCP incidence is half of that for RCP incidence, agreeing well with theoretical expectations. Figure 4(d) illustrates the simulated diffraction efficiency at different wavelengths. Here, the diffraction efficiency is defined as the ratio of the power of +1st or −2nd order to that of all diffraction orders. The average efficiencies for LCP and RCP incidences are ∼81 % and ∼87 % in the wavelength range of 9.3–10.6 μm, respectively, and the average efficiency for LP incidence reaches ∼84 %. For comparison, we also designed a spin-decoupled deflector based on the C2 meta-atoms selected in Figure 2, which has the same diffraction angle as the C3 metasurface (Supplementary Figures S4–S6). The average efficiencies for LCP and RCP incidences are ∼68 % and ∼62 % in the wavelength range of 9.3–10.6 μm, and the average efficiency for LP incidence is ∼64 %.

The experimental results of the deflector are shown in Figure 4(e). The optical setup and measurement procedures are presented in Section S4, Supplementary Material. Under LCP and RCP illumination, the cross-polarized RCP and LCP lights are diffracted to the +1st order and −2nd order in the broadband range of 9.3–10.6 μm, respectively. Under linear polarization (LP) illumination, the light is diffracted to the +1st order and −2nd order simultaneously. The measured efficiencies for LCP and RCP incidences reach ∼62 % and ∼63 % at 9.6 μm, respectively. To further demonstrate the broadband characteristics of the designed meta-device, we also measure the efficiencies at 9.3 μm, 10.2 μm, and 10.6 μm, marked by black dots in Figure 4(d). The linewidth of the CO_2_ laser is below 100 kHz, corresponding to spectral bandwidths of 0.029 pm, 0.031 pm, 0.035 pm, and 0.038 pm at 9.3 μm, 9.6 μm, 10.2 μm, and 10.6 μm, respectively. The measured efficiency is lower than the simulated results, which can be attributed to the fabrication and measurement errors (Supplementary Figure S3).

### 3.2 Demonstration of the spin-decoupled holograms

**Figure 5:**
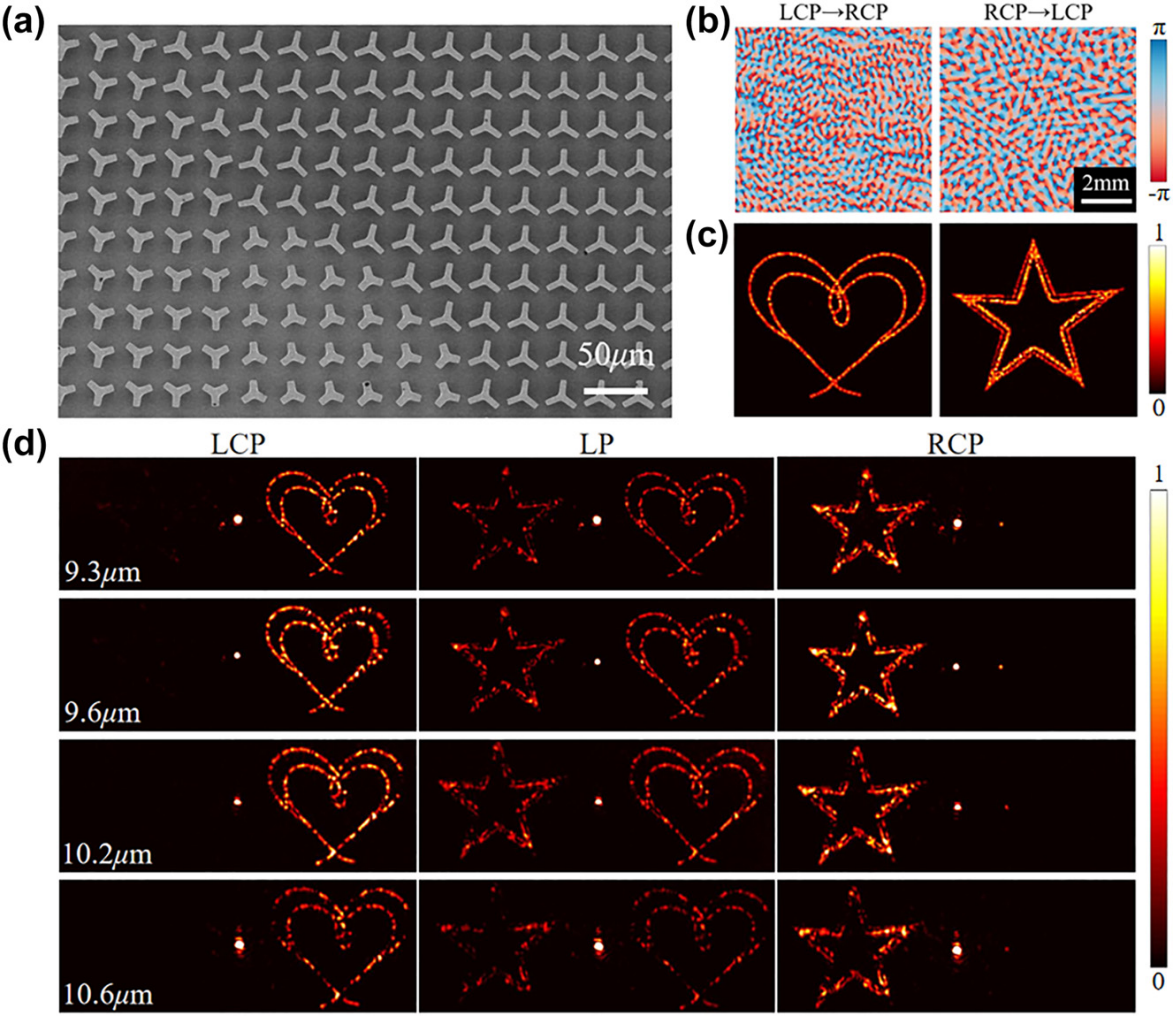
Spin-decoupled holograms with C3 meta-atoms. (a) SEM image of the fabricated meta-hologram device. (b) Required phase profiles to reconstruct the holographic images. (c) Simulated holographic images based on the phase profiles in (b). (d) Measured holographic images at different wavelengths under LCP, LP, and RCP illuminations, respectively.

A spin-decoupled metasurface hologram was designed and fabricated to demonstrate the complex phase modulation capability of the C3 meta-atoms. For LCP and RCP incidences, we set different target images, and then calculate the required phase maps of the holograms based on the Gerchberg–Saxton (GS) algorithm [36]. The holographic phase maps and reconstructed images are shown in Figure 5(b) and (c), respectively. To avoid overlapping of the holographic image and the 0th-order spot, a deflected phase is added to the target phase profiles. Then, the required propagation phase and orientation angles of each meta-atom can be calculated according to eqs. (3) and (4), and the corresponding structural parameters and in-plane rotation angle of each meta-atom can be determined. The SEM image of the spin-decoupled hologram is shown in Figure 5(a) (detailed SEM images of the sample are shown in Supplementary Figure S1). We conducted experimental tests at wavelengths of 9.3 μm, 9.6 μm, 10.2 μm, and 10.6 μm, with the results shown in Figure 5(d). Under LCP and RCP illumination, a heart-shaped diffraction pattern and a star-shaped diffraction pattern can be seen, respectively. Under LP illumination, two diffraction patterns can be seen at the same time. This shows the broadband modulation capability of the designed device, which agrees well with the theoretical expectation.

## 4 Materials and methods

### 4.1 Numerical simulations

Numerical simulations of the unit cells and the metasurface were performed using the finite element method with commercial software of COMSOL Multiphysics. The refractive index of the Au refers to Babar’s research in 2015 [37]. The periodic boundary conditions were applied in *x*- and *y*-directions in all simulations; the incident circularly polarized plane wave incident light was set to be incident from the positive *z*-direction in all simulations.

### 4.2 Sample fabrication

The process refers to our previous investigation [29], which involves spin-coating a silicon wafer with photoresist, patterning via lithography, etching the silicon, stripping the photoresist with oxygen, and depositing a gold layer through sputtering.

## 5 Conclusions

In summary, we have realized ASOI in C3 rotationally symmetric meta-atoms by merging the generalized PB geometric phase and the propagation phase. Since the anisotropy of the C3 meta-atoms is less sensitive to the shapes of the meta-atoms compared to C2 meta-atoms, obvious advantage for C3 meta-atoms for realizing high-efficiency ASOI is observed. The spin-decoupled beam deflector based on C3 meta-atoms can achieve an average diffraction efficiency of ∼84 % in the broadband range of 9.3–10.6 μm, which is ∼20 % higher than that of C2 meta-atoms with the same period and height. We experimentally demonstrated a spin-decoupled beam deflector and hologram meta-device based on C3 meta-atoms, which perform well in the wavelength range of 9.3–10.6 μm. The high-order composite phase metasurface proposed in this paper is an excellent candidate for applications in complex vector optical field manipulation, multi-channel holography, optical storage, and polarization imaging, which is expected to promote the further development of digital optics [38] and engineering optics 2.0 [39].

## Supplementary Material

Supplementary Material Details

## References

[1] Aravind P. K. (1992). A simple proof of Pancharatnam’s theorem. *Opt. Commun.*.

[2] Chen S., Liu W., Li Z., Cheng H., Tian J. (2020). Metasurface‐Empowered optical multiplexing and multifunction. *Adv. Mater.*.

[3] Li S. (2018). Multidimensional manipulation of photonic spin Hall effect with a single‐layer dielectric metasurface. *Adv. Opt. Mater.*.

[4] Luo X. (2015). Principles of electromagnetic waves in metasurfaces. *Sci. China: Phys., Mech. Astron.*.

[5] Lee G.-Y. (2017). Complete amplitude and phase control of light using broadband holographic metasurfaces. *Nanoscale*.

[6] Deng Z.-L. (2020). Full‐color complex‐amplitude vectorial holograms based on multi‐freedom metasurfaces. *Adv. Funct. Mater.*.

[7] Zhong F., Li J., Liu H., Zhu S.-N. (2018). Controlling surface plasmons through covariant transformation of the spin-dependent geometric phase between curved metamaterials. *Phys. Rev. Lett.*.

[8] Liu S., Chen S., Wen S., Luo H. (2022). Photonic spin Hall effect: fundamentals and emergent applications. *Opto-Electron. Sci.*.

[9] Litchinitser N. M. (2016). Photonic multitasking enabled with geometric phase. *Science*.

[10] Li Z. (2017). Dielectric meta-holograms enabled with dual magnetic resonances in visible light. *ACS Nano*.

[11] Lin D., Fan P., Hasman E., Brongersma M. L. (2014). Dielectric gradient metasurface optical elements. *Science*.

[12] Zheng G., Mühlenbernd H., Kenney M., Li G., Zentgraf T., Zhang S. (2015). Metasurface holograms reaching 80% efficiency. *Nat. Nanotechnol.*.

[13] Pu M. (2015). Catenary optics for achromatic generation of perfect optical angular momentum. *Sci. Adv.*.

[14] Huang Y. (2023). All-optical controlled-NOT logic gate achieving directional asymmetric transmission based on metasurface doublet. *Opto-Electron. Adv.*.

[15] Zeng C. (2022). Graphene-empowered dynamic metasurfaces and metadevices. *Opto-Electron. Adv.*.

[16] Zhang X. (2018). Polarization-independent broadband meta-holograms via polarization-dependent nanoholes. *Nanoscale*.

[17] Wen D. (2015). Helicity multiplexed broadband metasurface holograms. *Nat. Commun.*.

[18] Ma X. (2015). A planar chiral meta-surface for optical vortex generation and focusing. *Sci. Rep.*.

[19] Pu M., Guo Y., Li X., Ma X., Luo X. (2018). Revisitation of extraordinary young’s interference: from catenary optical fields to spin–orbit interaction in metasurfaces. *ACS Photonics*.

[20] Chen S. (2024). Towards the performance limit of catenary meta-optics via field-driven optimization. *Opto-Electron. Adv.*.

[21] Balthasar Mueller J. P., Rubin N. A., Devlin R. C., Groever B., Capasso F. (2017). Metasurface polarization optics: independent phase control of arbitrary orthogonal states of polarization. *Phys. Rev. Lett.*.

[22] Zhang F., Pu M., Luo J., Yu H., Luo X. (2017). Symmetry breaking of photonic spin-orbit interactions in metasurfaces. *Opto-Electron. Eng.*.

[23] Guo Y. (2016). Merging geometric phase and plasmon retardation phase in continuously shaped metasurfaces for arbitrary orbital angular momentum generation. *ACS Photonics*.

[24] Zhang F., Guo Y., Pu M., Li X., Ma X., Luo X. (2020). Metasurfaces enabled by asymmetric photonic spin-orbit interactions. *Opto-Electron. Eng.*.

[25] Zhang F., Cai J., Pu M., Luo X. (2021). Composite-phase manipulation in optical metasurfaces. *Physics*.

[26] Guo Y. (2021). Spin-decoupled metasurface for simultaneous detection of spin and orbital angular momenta via momentum transformation. *Light: Sci. Appl.*.

[27] Zhang F. (2023). Meta-optics empowered vector visual cryptography for high security and rapid decryption. *Nat. Commun.*.

[28] Zhang Y. (2022). Crosstalk-free achromatic full Stokes imaging polarimetry metasurface enabled by polarization-dependent phase optimization. *Opto-Electron. Adv.*.

[29] Cai J. (2022). All-metallic high-efficiency generalized Pancharatnam–Berry phase metasurface with chiral meta-atoms. *Nanophotonics*.

[30] Xie X. (2021). Generalized pancharatnam-berry phase in rotationally symmetric meta-atoms. *Phys. Rev. Lett.*.

[31] Ding X. (2015). Ultrathin pancharatnam–berry metasurface with maximal cross‐polarization efficiency. *Adv. Mater.*.

[32] Guo Y. (2022). Classical and generalized geometric phase in electromagnetic metasurfaces. *Photonics Insights*.

[33] Liu M. (2021). Broadband generation of perfect Poincaré beams via dielectric spin-multiplexed metasurface. *Nat. Commun.*.

[34] Chen S. (2021). Cylindrical vector beam multiplexer/demultiplexer using off-axis polarization control. *Light: Sci. Appl.*.

[35] Zhang S. (2020). Broadband detection of multiple spin and orbital angular momenta via dielectric metasurface. *Laser Photonics Rev.*.

[36] Gerchberg R. W., Saxton W. O. (1972). A practical algorithm for the determination of phase from image and diffraction plane pictures. *Optik*.

[37] Babar S., Weaver J. (2015). Optical constants of Cu, Ag, and Au revisited. *Appl. Opt.*.

[38] Capasso F. (2018). The future and promise of flat optics: a personal perspective. *Nanophotonics*.

[39] Luo X. (2018). Engineering optics 2.0: a revolution in optical materials, devices, and systems. *ACS Photonics*.

