# Supplementary material for: High-efficiency and broadband asymmetric spin–orbit interaction based on high-order composite phase modulation
Source: Nanophotonics. 2024 Sep 6;13(22):4203–10. doi: 10.1515/nanoph-2024-0344 (PMC11501051; doi:10.1515/nanoph-2024-0344)
Supplement: Supplementary file 1 — Supplementary Material Details [file j_nanoph-2024-0344_suppl_001.docx]

**Supplementary material**

High-Efficiency Asymmetric Spin-Orbit Interaction in High-Order Rotationally Symmetric Meta-atoms

*Yuzhong Ou^1,2,3,4^, Yan Chen^1,2,3,4^, Fei Zhang^1,2,3,4^, Mingbo Pu^1,2,3,4,*^, Mengna Jiang^1,2,3,4^, Mingfeng Xu^1,2,3,4^, Yinghui Guo^1,2,3,4^, Chaolong Feng^2,3,4^, Ping Gao^2,3,4^, and Xiangang Luo^1,2,3,4,*^*

1 National Key Laboratory of Optical Field Manipulation Science and Technology, Chinese Academy of Sciences, Chengdu 610209, China

2 State Key Laboratory of Optical Technologies on Nano-Fabrication and Micro-Engineering, Chengdu 610209, China

3 Institute of Optics and Electronics, Chinese Academy of Sciences, Chengdu 610209, China

4 College of Materials Sciences and Opto-Electronic Technology, University of Chinese Academy of Sciences, Beijing 100049, China

***E-mails: pmb@ioe.ac.cn; lxg@ioe.ac.cn**

**S1. The scanning electron microscope images of the sample**

Figure S1 shows the SEM images of the beam deflector and the holographic meta-device fabricated using the C3 meta-atoms. Figure S1(a) is the top view of the deflector when the scales are 50 *μ*m, 20 *μ*m and 10 *μ*m, and Figure S1(b) is the image of the deflector with a slight angle when the scales are 50 *μ*m, 20 *μ*m and 10 *μ*m. Figure S1(c) is the top view of the holographic device when the scale is 50 *μ*m, 20 *μ*m and 10 *μ*m, and Figure S1(d) is the image of the holographic device with a tilt angle when the scale is 50 *μ*m, 20 *μ*m and 10 *μ*m. Due to the high aspect ratio of meta-atoms, the sidewall will be slightly tilted by inductively coupled plasma etching. A 300 nm thick gold layer (larger than skin depth) is deposited on the Si wafer through magnetron sputtering [1], and the thickness of gold layers is not the same on the top and sidewalls [2].Additionally, the weak bond between the gold and silicon layers can lower meta-device efficiency, but this can be enhanced by inserting a chromium layer [2].


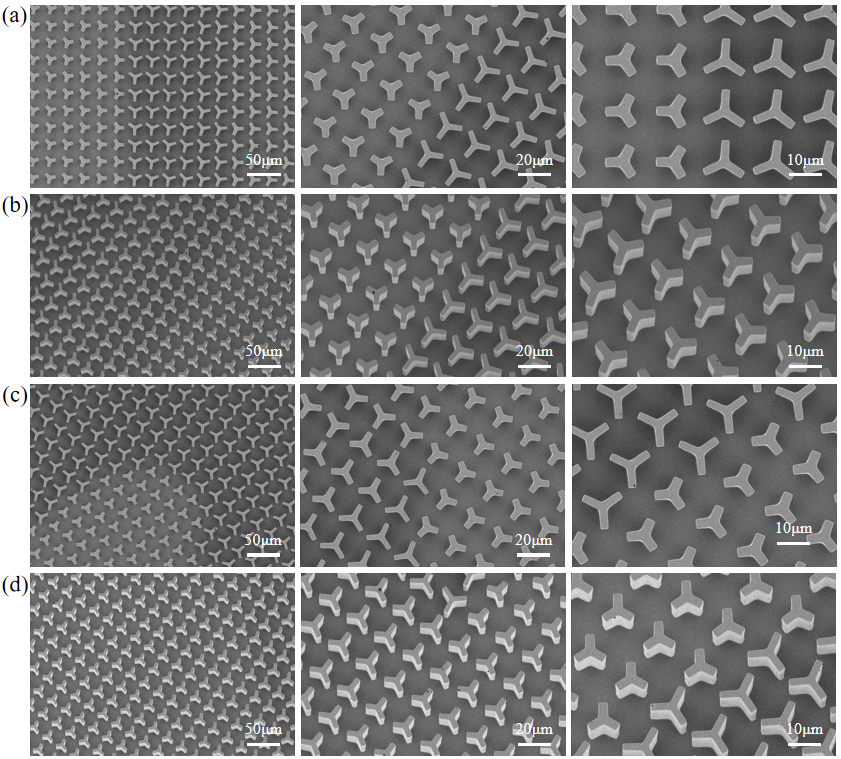


**Figure S1.** The SEM images of the sample. (a) The top view of the deflector when the scales are 50 *μ*m, 20 *μ*m and 10 *μ*m. (b) The image of the deflector with a slight angle when the scales are 50 *μ*m, 20 *μ*m and 10 *μ*m. (c) The top view of the holographic device when the scale is 50 *μ*m, 20 *μ*m and 10 *μ*m. (d) The image of the holographic device with a tilt angle when the scale is 50 *μ*m, 20 *μ*m and 10 *μ*m.

**S2. Simulated far-field intensity distribution of the beam deflectors based on C3 meta-atoms**

Figure S2 shows the simulated normalized far-field intensity distribution of the beam deflector based on C3 meta-atoms at the wavelength of 10 *μ*m. Perfect deflection behavior can be observed, the deflection angle for LCP incidence is -3° and the deflection angle for RCP incidence is 6°, which is coincident well with the theoretically designed value. The diffraction efficiency of LCP incidence is 87.2%, while that of RCP incidence is 81.1%. The deflection angle is designed to be small so that the diffraction orders can be received by the CCD in the measurements.

Figure S3 shows the simulated normalized far-field intensity distribution of the beam deflector based on C3 meta-atoms with a reduced linewidth of 0.5 *μ*m at the wavelength of 10 *μ*m. It can be seen that the deflection angle for LCP incidence is -3° and the deflection angle for RCP incidence is 6°. The diffraction efficiency of LCP incidence is 68.7%, while that of RCP incidence is 66.1%, which is poor compared to the beam deflector above.

**
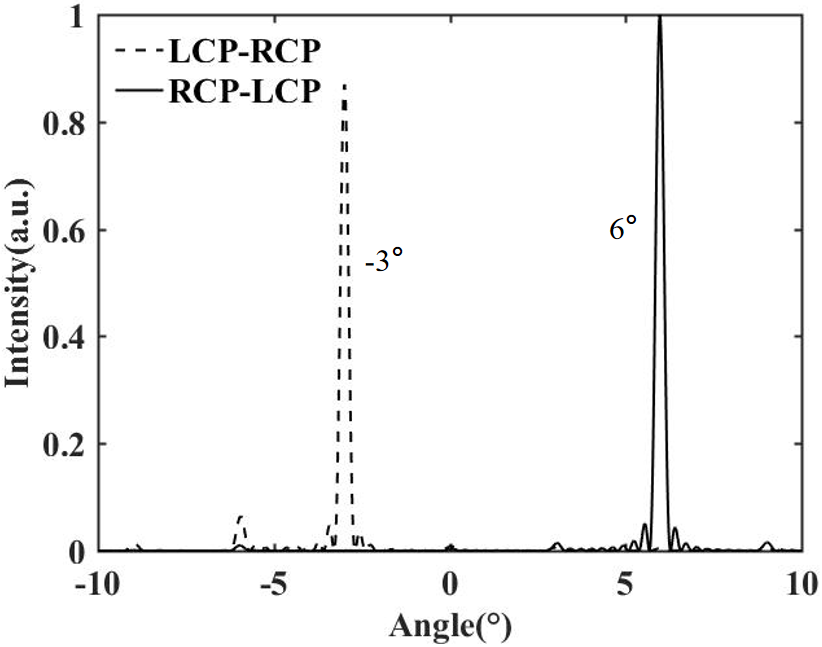
**

**Figure S2.** Simulated far-field intensity distribution of the beam deflector based on C3 meta-atoms under LCP and RCP normal incidence at the wavelength of 10 *μ*m.

**
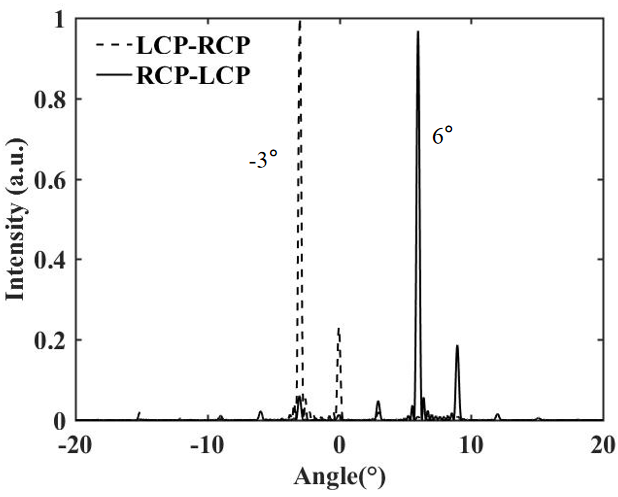
**

**Figure S3.** Simulated far-field intensity distribution of the beam deflector based on C3 meta-atoms with a reduced linewidth of 0.5 *μ*m under LCP and RCP normal incidence at the wavelength of 10 *μ*m.

**S3. The design of the beam deflector based on C2 meta-atoms**

A spin-decoupled beam deflector with C2 meta-atoms was designed, as shown in Figure S4. The deflector consists of periodically arranged supercells, which contain 24 meta-atoms with different sizes and rotation angles. The phase gradient between adjacent meta-atoms is designed as 15° and 30°, respectively for LCP and RCP incidence, and the corresponding deflection angles are 3° and 6°.

Figure S5 shows the simulated normalized far-field intensity distribution of the beam deflector based on C2 meta-atoms at the wavelength of 10 *μ*m. It can be seen that the deflection angle for LCP incidence is -3° and the deflection angle for RCP incidence is 6°. The diffraction efficiency is poor compared to the beam deflector based on C3 meta-atoms.

The simulated diffraction efficiency is shown in Figure S6. The average efficiencies for LCP and RCP incidence are ~68% and ~62% in the broadband range of 9.3-10.6 *μ*m, which are not as high as C3 meta-atoms.


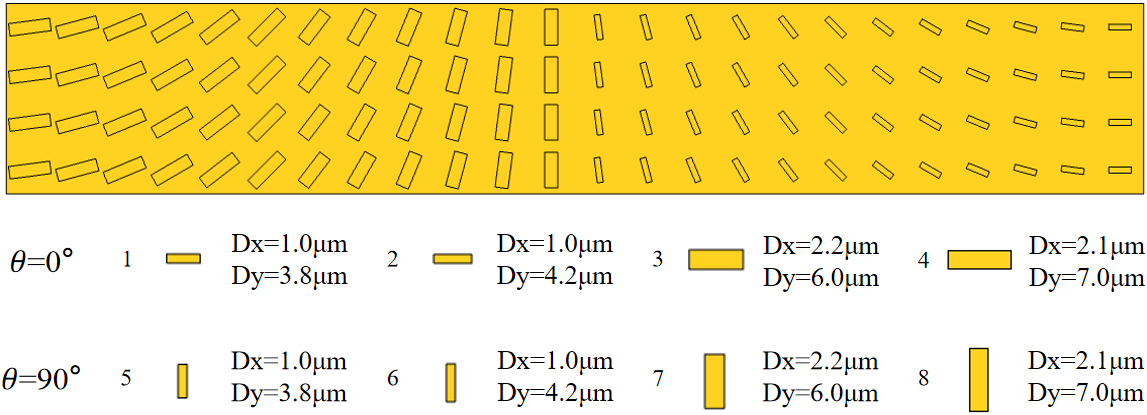


**Figure S4.** Spin-decoupled meta-deflector with C2 meta-atoms.

**
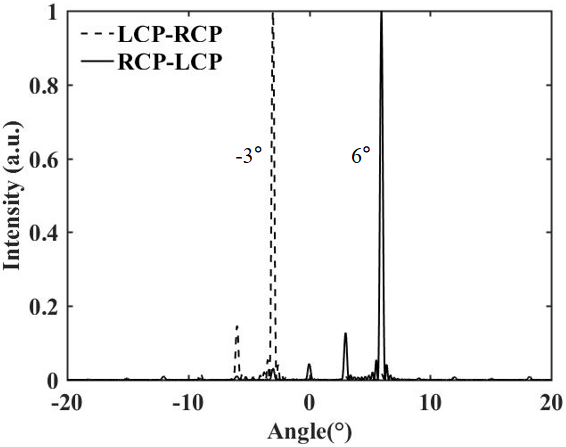
**

**Figure S5.** Simulated far-field intensity distribution of the beam deflector based on C2 meta-atoms under LCP and RCP normal incidence at the wavelength of 10 *μ*m.


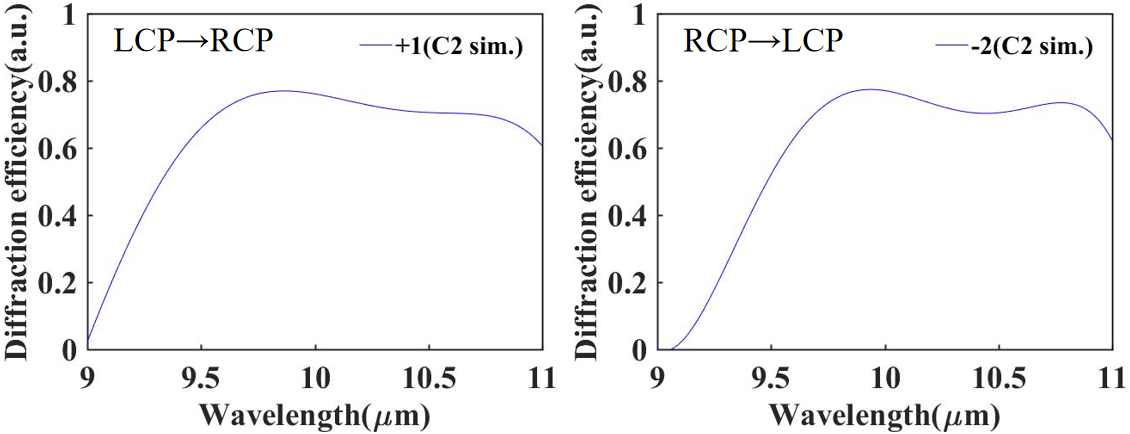


**Figure S6.** The diffraction efficiency of the simulation of the C2 deflector for LCP and RCP illumination.

**S4. Measurement method**

Figure S7 illustrates the schematic of the measurement setup. A CO_2_ laser was utilized as the light source. After passing through an adjustable attenuator, the light beam was sent through a beam expander, an adjustable aperture, a linear polarizer, a quarter wave-plate, another adjustable aperture, and then illuminated on the sample. A beam splitter was placed in front of the sample to guide the reflected light to an infrared charge-coupled device (CCD) (384 × 288 pixels, UA330, Guide-Infrared Inc.). The size of each pixel is 25 *μ*m × 25 *μ*m. There was a lens in front of the CCD for focusing, and the lens was removed during efficiency measurement.


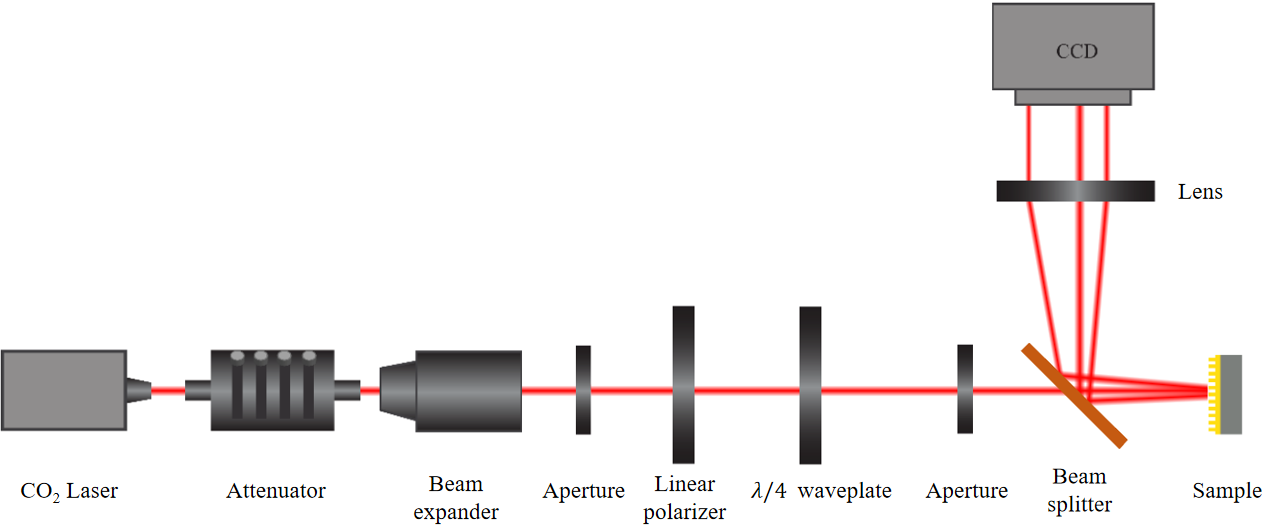


**Figure S7.** Schematic illustration of the measurement setup.

**S5. The absorption loss from the C3 metallic metasurface**

This paper perform a scan of the absorption loss from the C3 meta-atom at the wavelength of 10 *μ*m, as shown in Figure S8. Here, the absorption loss is defined as *A* = 1 - *R*, where *R* is the reflectivity. We also measured the absorption loss from the metallic film at 9.3 *μ*m, 9.6 *μ*m, 10.2 *μ*m, and 10.6 *μ*m, with the results being 0.039, 0.038, 0.047, and 0.048, respectively.


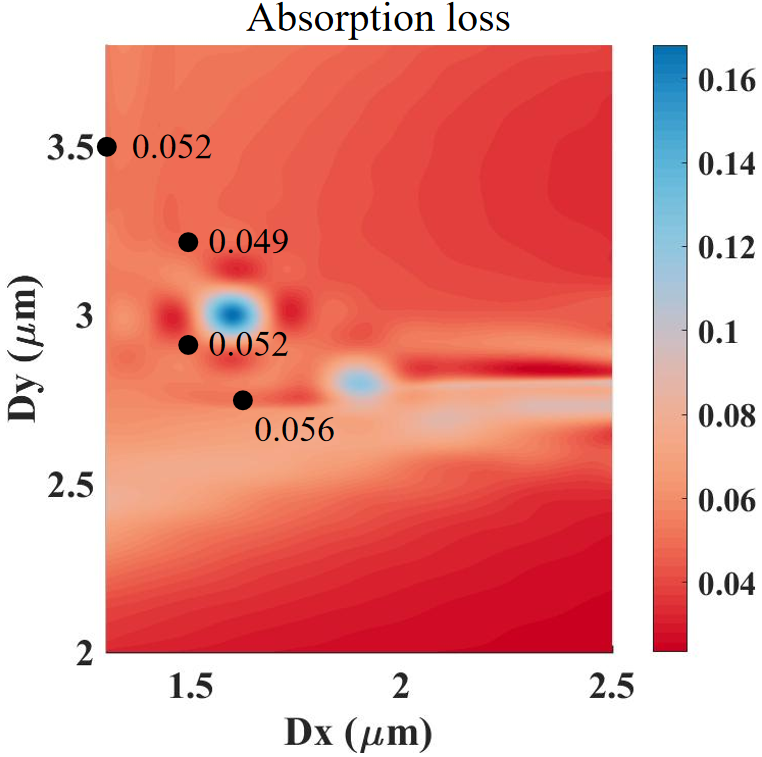


**Figure S8.** The absorption loss from the C3 meta-atom.

**S6. Comparison between C3 meta-atom and C5 meta-atom**

To investigate the efficacy of higher-order phase modulation, this paper conducts a parameter sweep of the C5 structure at a wavelength of 10 μm. The C5 structure model is shown in Figures S9(A). For comparison, the C5 structure has the same period, height, and linewidth range as the C3 structure. Simulated absorption loss, conversion efficiency, and propagation phase of C5 meta-atoms as a function of structural parameters are show in Figure S9(B) - (D). To achieve full 360° propagation phase coverage for the 8 structures, 4 fundamental structures covering approximately 180° phase range are selected, with the conversion efficiencies of approximately 0.764, 0.300, 0.012 and 0.001, respectively. And the absorption losses of 4 fundamental structures are approximately 0.224, 0.150, 0.045, and 0.028, respectively. It can be seen that within the gray dashed region (where the linewidth of the C5 meta-atom exceeds 1 μm), it is challenging to find a set of structures with high efficiency that meet the phase coverage of 360°. This is because the anisotropy of the C5 meta-atoms arranged in the square lattice is too weak, which results in low conversion efficiency. Therefore, for all-metallic configurations, C3 meta-atom is a better choice to achieve high-efficiency and easy-to-fabricate ASOI meta-devices than C5 meta-atom.


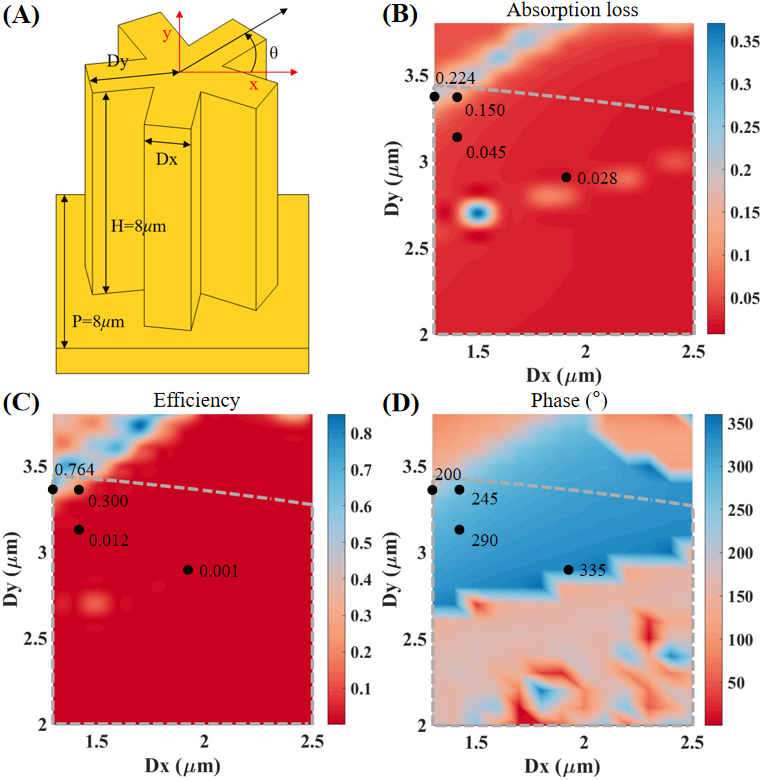


**Figure S9.** Comparison with C5 meta-atoms.

(A) C5 structure model. Simulated (B) absorption loss, (C) conversion efficiency, and (D) propagation phase of C5 meta-atoms as a function of structural parameters.

**References**

[1] J. Cai, et al., “All-metallic high-efficiency generalized Pancharatnam–Berry phase metasurface with chiral meta-atoms,” Nanophotonics, vol. 11, no. 9, pp. 1961-1968, 2022.

[2] X. Xie, et al., “All-metallic geometric metasurfaces for broadband and high-efficiency wavefront manipulation,” Nanophotonics, vol. 9, pp. 3209 - 3215, 2019.
